# Supplementary material for: Alfalfa leaf weevil larvae and adults feeding induces physiological change in defensive enzymes of alfalfa
Source: PLoS One. 2024 Nov 8;19(11):e0312612. doi: 10.1371/journal.pone.0312612 (PMC11548782; doi:10.1371/journal.pone.0312612)
Supplement: S1 File — (DOCX) [file pone.0312612.s001.docx]

**Supporting information**

Alfalfa leaf weevil larvae and adults feeding induces physiological change in alfalfa enzymes

Liu Hui^1,2^, Wang Xuzhe^1^, Ma Yong^1^, Gao Wanshun^1^, Ma Chunhui^1*^

1 College of Animal Science and Technology, Shihezi University, Shihezi, Xinjiang 832003, China;

2 College of Life Science, Shihezi University, Shihezi, Xinjiang 832003, China.

**Contents**

[1. CAT 1](#_Toc162536263)

[2. POD 3](#_Toc162536264)

[3. SOD 5](#_Toc162536265)

[4. T-AOC 7](#_Toc162536266)

[5. MDA 9](#_Toc162536267)

[6. PAL 11](#_Toc162536268)

[7. PPO 13](#_Toc162536269)

[8. TAL 15](#_Toc162536270)

[9. LOX 17](#_Toc162536271)

[10. CI 19](#_Toc162536272)

[11. TI 21](#_Toc162536273)

[12. JA 23](#_Toc162536274)

**1. CAT**

The kit assay Plant CAT level in the sample, use Purified Plant CAT antibody to coat microtiter plate wells, make solid-phase antibody, then add CAT to wells. Combined CAT antibody which With HRP labeled, become antibody-antigen-enzyme-antibody complex, after washing completely. Add TMB substrate solution, TMB substrate becomes blue color At HRP enzyme-catalyzed, reaction is terminated by the addition of a sulphuric acid solution and the color change is measured spectrophotometrically at a wavelength of 450 nm. The concentration of Plant CAT in the samples is then determined by comparing the OD of the samples to the standard curve.

Detection range, 0.3 U/mL~9U/mL.

Table S1 Materials provided with the kit

| No. | Reagents | Specifications | No. | Reagents | Specifications |
| --- | --- | --- | --- | --- | --- |
| 1 | wash solution | 20mL×1 bottle | 7 | Stop Solution | 6mL×1 bottle |
| 2 | HRP-Conjugate reagent | 6mL×1 bottle | 8 | Standard (16U/mL) | 0.5mL×1 bottle |
| 3 | Microelisa stripplate | 12well×8strips | 9 | Standard diluent | 1.5mL×1 bottle |
| 4 | Sample diluent | 6mL×1 bottle | 10 | Instruction | 1 |
| 5 | Chromogen Solution A | 6mL×1 bottle | 11 | Closure plate membrane | 2 |
| 6 | Chromogen Solution B | 6mL×1 bottle | 12 | Sealed bags | 1 |

1.1 Assay procedure

(1) Preparation the standard solutions according to Table S2.

Table S2 Preparation of standard solution

| Concentration | No. | Procedure |
| --- | --- | --- |
| 8U/mL | 5 Standard | 150μL Original density Standard +150μL Standard diluent |
| 4U/mL | 4 Standard | 150μL 5 Standard +150μL Standard diluent |
| 2U/mL | 3 Standard | 150μL 4 Standard +150μL Standard diluent |
| 1U/mL | 2 Standard | 150μL 3 Standard +150μL Standard diluent |
| 0.5U/mL | 1 Standard | 150μL 2 Standard +150μL Standard diluent |

(2) Add sample

On the Microelisa stripplate, blank wells (blank control wells without samples or ELISA reagents, the rest of the steps are the same), standard wells, and wells for the sample to be tested are set separately. Accurately add 50 μL of each concentration standard solution to the standard wells. Add 40 μL of sample diluent to the sample wells, and then add 10 μL of the sample to be tested (the final dilution of the sample is 5 times).

(3) Incubate

After closing plate with Closure plate membrane, incubated for 30 min at 37℃.

(4) Configurate liquid

The wash solution diluted 30-fold with distilled water.

(5) Washing

Uncovered closure plate membrane, discard Liquid, dry by swing, add washing buffer to every well, still for 30s then drain. Repeat those process 5 times and pat dry.

(6) Add enzyme

Add HRP-Conjugate reagent 50μL to each well, except the blank well.

(7) Incubate

Same as procedure 3.

(8) Washing

Same as procedure 3.

(9) Color rendering

Add Chromogen Solution A 50μL and Chromogen Solution B to each well, evade the light preservation for 10 min at 37℃.

(10) Stop the reaction

Add Stop Solution 50μL to each well, at which point the blue color changed to yellow color.

(11) Determination

Take blank well as zero, read the absorbance (OD value) of each well at 450nm. The measurement should be conducted within 15 min after adding the stop solution.

1.2 Calculate

Draw a standard curve with the concentration of the standard substance as the x-axis and the OD value as the y-axis. Then, the linear regression equation (y=778.16x+3.8757, R^2^=0.9957) of the standard curve is calculated using the concentration and OD value of the standard substance. The OD value of the sample is substituted into the equation to calculate the sample concentration, which is then multiplied by the dilution factor to obtain the actual concentration of the sample.

Figure S1 The standard curve of CAT

**2. POD**

The kit assay Plant POD level in the sample, use Purified Plant POD antibody to coat microtiter plate wells, make solid-phase antibody, then add POD to wells. Combined POD antibody which With HRP labeled, become antibody-antigen-enzyme-antibody complex, after washing completely. Add TMB substrate solution, TMB substrate becomes blue color At HRP enzyme-catalyzed, reaction is terminated by the addition of a sulphuric acid solution and the color change is measured spectrophotometrically at a wavelength of 450 nm. The concentration of Plant POD in the samples is then determined by comparing the OD of the samples to the standard curve.

Detection range, 0.6 mU/L~28 mU/L.

Table S3 Materials provided with the kit

| No. | Reagents | Specifications | No. | Reagents | Specifications |
| --- | --- | --- | --- | --- | --- |
| 1 | wash solution | 20mL×1 bottle | 7 | Stop Solution | 6mL×1 bottle |
| 2 | HRP-Conjugate reagent | 6mL×1 bottle | 8 | Standard (48mU/L) | 0.5mL×1 bottle |
| 3 | Microelisa stripplate | 12well×8strips | 9 | Standard diluent | 1.5mL×1 bottle |
| 4 | Sample diluent | 6mL×1 bottle | 10 | Instruction | 1 |
| 5 | Chromogen Solution A | 6mL×1 bottle | 11 | Closure plate membrane | 2 |
| 6 | Chromogen Solution B | 6mL×1 bottle | 12 | Sealed bags | 1 |

2.1 Assay procedure

(1) Preparation the standard solutions according to Table S4.

Table S4 Preparation of standard solution

| Concentration | No. | Procedure |
| --- | --- | --- |
| 24mU/L | 5 Standard | 150μL Original density Standard +150μL Standard diluent |
| 12mU/L | 4 Standard | 150μL 5 Standard +150μL Standard diluent |
| 6mU/L | 3 Standard | 150μL 4 Standard +150μL Standard diluent |
| 3mU/L | 2 Standard | 150μL 3 Standard +150μL Standard diluent |
| 1.5mU/L | 1 Standard | 150μL 2 Standard +150μL Standard diluent |

(2) Add sample

On the Microelisa stripplate, blank wells (blank control wells without samples or ELISA reagents, the rest of the steps are the same), standard wells, and wells for the sample to be tested are set separately. Accurately add 50 μL of each concentration standard solution to the standard wells. Add 40 μL of sample diluent to the sample wells, and then add 10 μL of the sample to be tested (the final dilution of the sample is 5 times).

(3) Incubate

After closing plate with Closure plate membrane, incubated for 30 min at 37℃.

(4) Configurate liquid

The wash solution diluted 30-fold with distilled water.

(5) Washing

Uncovered closure plate membrane, discard Liquid, dry by swing, add washing buffer to every well, still for 30s then drain. Repeat those process 5 times and pat dry.

(6) Add enzyme

Add HRP-Conjugate reagent 50μL to each well, except the blank well.

(7) Incubate

Same as procedure 3.

(8) Washing

Same as procedure 3.

(9) Color rendering

Add Chromogen Solution A 50μL and Chromogen Solution B to each well, evade the light preservation for 10 min at 37℃.

(10) Stop the reaction

Add Stop Solution 50μL to each well, at which point the blue color changed to yellow color.

(11) Determination

Take blank well as zero, read the absorbance (OD value) of each well at 450nm. The measurement should be conducted within 15 min after adding the stop solution.

2.2 Calculate

Draw a standard curve with the concentration of the standard substance as the x-axis and the OD value as the y-axis. Then, the linear regression equation (y=2.2472x+0.0389, R^2^=0.9965) of the standard curve is calculated using the concentration and OD value of the standard substance. The OD value of the sample is substituted into the equation to calculate the sample concentration, which is then multiplied by the dilution factor to obtain the actual concentration of the sample.

Figure S2 The standard curve of POD

**3. SOD**

The kit assay Plant SOD level in the sample, use Purified Plant SOD antibody to coat microtiter plate wells, make solid-phase antibody, then add SOD to wells. Combined SOD antibody which With HRP labeled, become antibody-antigen-enzyme-antibody complex, after washing completely. Add TMB substrate solution, TMB substrate becomes blue color At HRP enzyme-catalyzed, reaction is terminated by the addition of a sulphuric acid solution and the color change is measured spectrophotometrically at a wavelength of 450 nm. The concentration of Plant POD in the samples is then determined by comparing the OD of the samples to the standard curve.

Detection range, 120U/L~4200 U/L.

Table S5 Materials provided with the kit

| No. | Reagents | Specifications | No. | Reagents | Specifications |
| --- | --- | --- | --- | --- | --- |
| 1 | wash solution | 20mL×1 bottle | 7 | Stop Solution | 6mL×1 bottle |
| 2 | HRP-Conjugate reagent | 6mL×1 bottle | 8 | Standard (8000U/L) | 0.5mL×1 bottle |
| 3 | Microelisa stripplate | 12well×8strips | 9 | Standard diluent | 1.5mL×1 bottle |
| 4 | Sample diluent | 6mL×1 bottle | 10 | Instruction | 1 |
| 5 | Chromogen Solution A | 6mL×1 bottle | 11 | Closure plate membrane | 2 |
| 6 | Chromogen Solution B | 6mL×1 bottle | 12 | Sealed bags | 1 |

3.1 Assay procedure

(1) Preparation the standard solutions according to Table S6.

Table S6 Preparation of standard solution

| Concentration | No. | Procedure |
| --- | --- | --- |
| 4000U/L | 5 Standard | 150μL Original density Standard +150μL Standard diluent |
| 2000U/L | 4 Standard | 150μL 5 Standard +150μL Standard diluent |
| 1000U/L | 3 Standard | 150μL 4 Standard +150μL Standard diluent |
| 500U/L | 2 Standard | 150μL 3 Standard +150μL Standard diluent |
| 250U/L | 1 Standard | 150μL 2 Standard +150μL Standard diluent |

(2) Add sample

On the Microelisa stripplate, blank wells (blank control wells without samples or ELISA reagents, the rest of the steps are the same), standard wells, and wells for the sample to be tested were set separately. Accurately add 50 μL of each concentration standard solution to the standard wells. Add 40 μL of sample diluent to the sample wells, and then add 10 μL of the sample to be tested (the final dilution of the sample was 5 times).

(3) Incubate

After closing plate with Closure plate membrane, incubated for 30 min at 37℃.

(4) Configurate liquid

The wash solution diluted 30-fold with distilled water.

(5) Washing

Uncovered closure plate membrane, discard Liquid, dry by swing, add washing buffer to every well, still for 30s then drain. Repeat those process 5 times and pat dry.

(6) Add enzyme

Add HRP-Conjugate reagent 50μL to each well, except the blank well.

(7) Incubate

Same as procedure 3.

(8) Washing

Same as procedure 3.

(9) Color rendering

Add Chromogen Solution A 50μL and Chromogen Solution B to each well, evade the light preservation for 10 min at 37℃.

(10) Stop the reaction

Add Stop Solution 50μL to each well, at which point the blue color changed to yellow color.

(11) Determination

Take blank well as zero, read the absorbance (OD value) of each well at 450nm. The measurement should be conducted within 15 min after adding the stop solution.

3.2 Calculate

Draw a standard curve with the concentration of the standard substance as the x-axis and the OD value as the y-axis. Then, the linear regression equation (y=451.71x-5.1004, R^2^=0.9962) of the standard curve is calculated using the concentration and OD value of the standard substance. The OD value of the sample is substituted into the equation to calculate the sample concentration, which is then multiplied by the dilution factor to obtain the actual concentration of the sample.

Figure S3 The standard curve of SOD

**4. T-AOC**

The kit assay Plant T-AOC level in the sample, use Purified Plant T-AOC antibody to coat microtiter plate wells, make solid-phase antibody, then add T-AOC to wells. Combined T-AOC antibody which With HRP labeled, become antibody-antigen-enzyme-antibody complex, after washing completely. Add TMB substrate solution, TMB substrate becomes blue color At HRP enzyme-catalyzed, reaction is terminated by the addition of a sulphuric acid solution and the color change is measured spectrophotometrically at a wavelength of 450 nm. The concentration of Plant T-AOC in the samples is then determined by comparing the OD of the samples to the standard curve.

Detection range, 2pmol/L~90pmol/L.

Table S7 Materials provided with the kit

| No. | Reagents | Specifications | No. | Reagents | Specifications |
| --- | --- | --- | --- | --- | --- |
| 1 | wash solution | 20mL×1 bottle | 7 | Stop Solution | 6mL×1 bottle |
| 2 | HRP-Conjugate reagent | 6mL×1 bottle | 8 | Standard (160pmol/L) | 0.5mL×1 bottle |
| 3 | Microelisa stripplate | 12well×8strips | 9 | Standard diluent | 1.5mL×1 bottle |
| 4 | Sample diluent | 6mL×1 bottle | 10 | Instruction | 1 |
| 5 | Chromogen Solution A | 6mL×1 bottle | 11 | Closure plate membrane | 2 |
| 6 | Chromogen Solution B | 6mL×1 bottle | 12 | Sealed bags | 1 |

4.1 Assay procedure

(1) Preparation the standard solutions according to Table S8.

Table S8 Preparation of standard solution

| Concentration | No. | Procedure |
| --- | --- | --- |
| 80pmol/L | 5 Standard | 150μL Original density Standard +150μL Standard diluent |
| 40pmol/L | 4 Standard | 150μL 5 Standard +150μL Standard diluent |
| 20pmol/L | 3 Standard | 150μL 4 Standard +150μL Standard diluent |
| 10pmol/L | 2 Standard | 150μL 3 Standard +150μL Standard diluent |
| 5pmol/L | 1 Standard | 150μL 2 Standard +150μL Standard diluent |

(2) Add sample

On the Microelisa stripplate, blank wells (blank control wells without samples or ELISA reagents, the rest of the steps are the same), standard wells, and wells for the sample to be tested were set separately. Accurately add 50 μL of each concentration standard solution to the standard wells. Add 40 μL of sample diluent to the sample wells, and then add 10 μL of the sample to be tested (the final dilution of the sample was 5 times).

(3) Incubate

After closing plate with Closure plate membrane, incubated for 30 min at 37℃.

(4) Configurate liquid

The wash solution diluted 30-fold with distilled water.

(5) Washing

Uncovered closure plate membrane, discard Liquid, dry by swing, add washing buffer to every well, still for 30s then drain. Repeat those process 5 times and pat dry.

(6) Add enzyme

Add HRP-Conjugate reagent 50μL to each well, except the blank well.

(7) Incubate

Same as procedure 3.

(8) Washing

Same as procedure 3.

(9) Color rendering

Add Chromogen Solution A 50μL and Chromogen Solution B to each well, evade the light preservation for 10 min at 37℃.

(10) Stop the reaction

Add Stop Solution 50μL to each well, at which point the blue color changed to yellow color.

(11) Determination

Take blank well as zero, read the absorbance (OD value) of each well at 450nm. The measurement should be conducted within 15 min after adding the stop solution.

4.2 Calculate

Draw a standard curve with the concentration of the standard substance as the x-axis and the OD value as the y-axis. Then, the linear regression equation (y=8.028x+0.0763, R^2^=0.9963) of the standard curve is calculated using the concentration and OD value of the standard substance. The OD value of the sample is substituted into the equation to calculate the sample concentration, which is then multiplied by the dilution factor to obtain the actual concentration of the sample.

Figure S4 The standard curve of T-AOC

**5. MDA**

The kit assay Plant MDA level in the sample, use Purified Plant MDA antibody to coat microtiter plate wells, make solid-phase antibody, then add MDA to wells. Combined MDA antibody which With HRP labeled, become antibody-antigen-enzyme-antibody complex, after washing completely. Add TMB substrate solution, TMB substrate becomes blue color At HRP enzyme-catalyzed, reaction is terminated by the addition of a sulphuric acid solution and the color change is measured spectrophotometrically at a wavelength of 450 nm. The concentration of Plant MDA in the samples is then determined by comparing the OD of the samples to the standard curve.

Detection range, 0.2 nmol/L~4.8 nmol/L.

Table S9 Materials provided with the kit

| No. | Reagents | Specifications | No. | Reagents | Specifications |
| --- | --- | --- | --- | --- | --- |
| 1 | wash solution | 20mL×1 bottle | 7 | Stop Solution | 6mL×1 bottle |
| 2 | HRP-Conjugate reagent | 6mL×1 bottle | 8 | Standard (9.6 nmol/L) | 0.5mL×1 bottle |
| 3 | Microelisa stripplate | 12well×8strips | 9 | Standard diluent | 1.5mL×1 bottle |
| 4 | Sample diluent | 6mL×1 bottle | 10 | Instruction | 1 |
| 5 | Chromogen Solution A | 6mL×1 bottle | 11 | Closure plate membrane | 2 |
| 6 | Chromogen Solution B | 6mL×1 bottle | 12 | Sealed bags | 1 |

5.1 Assay procedure

(1) Preparation the standard solutions according to Table S10.

Table S10 Preparation of standard solution

| Concentration | No. | Procedure |
| --- | --- | --- |
| 4.8 nmol/L | 5 Standard | 150μL Original density Standard +150μL Standard diluent |
| 2.4 nmol/L | 4 Standard | 150μL 5 Standard +150μL Standard diluent |
| 1.2 nmol/L | 3 Standard | 150μL 4 Standard +150μL Standard diluent |
| 0.6 nmol/L | 2 Standard | 150μL 3 Standard +150μL Standard diluent |
| 0.3 nmol/L | 1 Standard | 150μL 2 Standard +150μL Standard diluent |

(2) Add sample

On the Microelisa stripplate, blank wells (blank control wells without samples or ELISA reagents, the rest of the steps are the same), standard wells, and wells for the sample to be tested were set separately. Accurately add 50 μL of each concentration standard solution to the standard wells. Add 40 μL of sample diluent to the sample wells, and then add 10 μL of the sample to be tested (the final dilution of the sample was 5 times).

(3) Incubate

After closing plate with Closure plate membrane, incubated for 30 min at 37℃.

(4) Configurate liquid

The wash solution diluted 30-fold with distilled water.

(5) Washing

Uncovered closure plate membrane, discard Liquid, dry by swing, add washing buffer to every well, still for 30s then drain. Repeat those process 5 times and pat dry.

(6) Add enzyme

Add HRP-Conjugate reagent 50μL to each well, except the blank well.

(7) Incubate

Same as procedure 3.

(8) Washing

Same as procedure 3.

(9) Color rendering

Add Chromogen Solution A 50μL and Chromogen Solution B to each well, evade the light preservation for 10 min at 37℃.

(10) Stop the reaction

Add Stop Solution 50μL to each well, at which point the blue color changed to yellow color.

(11) Determination

Take blank well as zero, read the absorbance (OD value) of each well at 450nm. The measurement should be conducted within 15 min after adding the stop solution.

5.2 Calculate

Draw a standard curve with the concentration of the standard substance as the x-axis and the OD value as the y-axis. Then, the linear regression equation (y=0.478x+0.0017, R^2^=0.9964) of the standard curve is calculated using the concentration and OD value of the standard substance. The OD value of the sample is substituted into the equation to calculate the sample concentration, which is then multiplied by the dilution factor to obtain the actual concentration of the sample.

Figure S5 The standard curve of MDA

**6. PAL**

The kit assay Plant PAL level in the sample, use Purified Plant PAL antibody to coat microtiter plate wells, make solid-phase antibody, then add PAL to wells. Combined PAL antibody which With HRP labeled, become antibody-antigen-enzyme-antibody complex, after washing completely. Add TMB substrate solution, TMB substrate becomes blue color At HRP enzyme-catalyzed, reaction is terminated by the addition of a sulphuric acid solution and the color change is measured spectrophotometrically at a wavelength of 450 nm. The concentration of Plant PAL in the samples is then determined by comparing the OD of the samples to the standard curve.

Detection range, 1.3U/L~40 U/L.

Table S11 Materials provided with the kit

| No. | Reagents | Specifications | No. | Reagents | Specifications |
| --- | --- | --- | --- | --- | --- |
| 1 | wash solution | 20mL×1 bottle | 7 | Stop Solution | 6mL×1 bottle |
| 2 | HRP-Conjugate reagent | 6mL×1 bottle | 8 | Standard (80U/L) | 0.5mL×1 bottle |
| 3 | Microelisa stripplate | 12well×8strips | 9 | Standard diluent | 1.5mL×1 bottle |
| 4 | Sample diluent | 6mL×1 bottle | 10 | Instruction | 1 |
| 5 | Chromogen Solution A | 6mL×1 bottle | 11 | Closure plate membrane | 2 |
| 6 | Chromogen Solution B | 6mL×1 bottle | 12 | Sealed bags | 1 |

6.1 Assay procedure

(1) Preparation the standard solutions according to Table S12.

Table S12 Preparation of standard solution

| Concentration | No. | Procedure |
| --- | --- | --- |
| 40 U/L | 5 Standard | 150μL Original density Standard +150μL Standard diluent |
| 20 U/L | 4 Standard | 150μL 5 Standard +150μL Standard diluent |
| 10 U/L | 3 Standard | 150μL 4 Standard +150μL Standard diluent |
| 5 U/L | 2 Standard | 150μL 3 Standard +150μL Standard diluent |
| 2.5 U/L | 1 Standard | 150μL 2 Standard +150μL Standard diluent |

(2) Add sample

On the Microelisa stripplate, blank wells (blank control wells without samples or ELISA reagents, the rest of the steps are the same), standard wells, and wells for the sample to be tested were set separately. Accurately add 50 μL of each concentration standard solution to the standard wells. Add 40 μL of sample diluent to the sample wells, and then add 10 μL of the sample to be tested (the final dilution of the sample was 5 times).

(3) Incubate

After closing plate with Closure plate membrane, incubated for 30 min at 37℃.

(4) Configurate liquid

The wash solution diluted 30-fold with distilled water.

(5) Washing

Uncovered closure plate membrane, discard Liquid, dry by swing, add washing buffer to every well, still for 30s then drain. Repeat those process 5 times and pat dry.

(6) Add enzyme

Add HRP-Conjugate reagent 50μL to each well, except the blank well.

(7) Incubate

Same as procedure 3.

(8) Washing

Same as procedure 3.

(9) Color rendering

Add Chromogen Solution A 50μL and Chromogen Solution B to each well, evade the light preservation for 10 min at 37℃.

(10) Stop the reaction

Add Stop Solution 50μL to each well, at which point the blue color changed to yellow color.

(11) Determination

Take blank well as zero, read the absorbance (OD value) of each well at 450nm. The measurement should be conducted within 15 min after adding the stop solution.

6.2 Calculate

Draw a standard curve with the concentration of the standard substance as the x-axis and the OD value as the y-axis. Then, the linear regression equation (y=4.1249x+0.0849, R^2^=0.9959) of the standard curve is calculated using the concentration and OD value of the standard substance. The OD value of the sample is substituted into the equation to calculate the sample concentration, which is then multiplied by the dilution factor to obtain the actual concentration of the sample.

Figure S6 The standard curve of PAL

**7. PPO**

The kit assay Plant PPO level in the sample, use Purified Plant PPO antibody to coat microtiter plate wells, make solid-phase antibody, then add PPO to wells. Combined PPO antibody which With HRP labeled, become antibody-antigen-enzyme-antibody complex, after washing completely. Add TMB substrate solution, TMB substrate becomes blue color At HRP enzyme-catalyzed, reaction is terminated by the addition of a sulphuric acid solution and the color change is measured spectrophotometrically at a wavelength of 450 nm. The concentration of Plant PPO in the samples is then determined by comparing the OD of the samples to the standard curve.

Detection range, 3.5IU/L~150IU/L.

Table S13 Materials provided with the kit

| No. | Reagents | Specifications | No. | Reagents | Specifications |
| --- | --- | --- | --- | --- | --- |
| 1 | wash solution | 20mL×1 bottle | 7 | Stop Solution | 6mL×1 bottle |
| 2 | HRP-Conjugate reagent | 6mL×1 bottle | 8 | Standard (240 IU/L) | 0.5mL×1 bottle |
| 3 | Microelisa stripplate | 12well×8strips | 9 | Standard diluent | 1.5mL×1 bottle |
| 4 | Sample diluent | 6mL×1 bottle | 10 | Instruction | 1 |
| 5 | Chromogen Solution A | 6mL×1 bottle | 11 | Closure plate membrane | 2 |
| 6 | Chromogen Solution B | 6mL×1 bottle | 12 | Sealed bags | 1 |

7.1 Assay procedure

(1) Preparation the standard solutions according to Table S14.

Table S14 Preparation of standard solution

| Concentration | No. | Procedure |
| --- | --- | --- |
| 120 IU/L | 5 Standard | 150μL Original density Standard +150μL Standard diluent |
| 60 IU/L | 4 Standard | 150μL 5 Standard +150μL Standard diluent |
| 30 IU/L | 3 Standard | 150μL 4 Standard +150μL Standard diluent |
| 15 IU/L | 2 Standard | 150μL 3 Standard +150μL Standard diluent |
| 7.5 IU/L | 1 Standard | 150μL 2 Standard +150μL Standard diluent |

(2) Add sample

On the Microelisa stripplate, blank wells (blank control wells without samples or ELISA reagents, the rest of the steps are the same), standard wells, and wells for the sample to be tested were set separately. Accurately add 50 μL of each concentration standard solution to the standard wells. Add 40 μL of sample diluent to the sample wells, and then add 10 μL of the sample to be tested (the final dilution of the sample was 5 times).

(3) Incubate

After closing plate with Closure plate membrane, incubated for 30 min at 37℃.

(4) Configurate liquid

The wash solution diluted 30-fold with distilled water.

(5) Washing

Uncovered closure plate membrane, discard Liquid, dry by swing, add washing buffer to every well, still for 30s then drain. Repeat those process 5 times and pat dry.

(6) Add enzyme

Add HRP-Conjugate reagent 50μL to each well, except the blank well.

(7) Incubate

Same as procedure 3.

(8) Washing

Same as procedure 3.

(9) Color rendering

Add Chromogen Solution A 50μL and Chromogen Solution B to each well, evade the light preservation for 10 min at 37℃.

(10) Stop the reaction

Add Stop Solution 50μL to each well, at which point the blue color changed to yellow color.

(11) Determination

Take blank well as zero, read the absorbance (OD value) of each well at 450nm. The measurement should be conducted within 15 min after adding the stop solution.

7.2 Calculate

Draw a standard curve with the concentration of the standard substance as the x-axis and the OD value as the y-axis. Then, the linear regression equation (y=13.155x-0.1419, R^2^=0.9956) of the standard curve is calculated using the concentration and OD value of the standard substance. The OD value of the sample is substituted into the equation to calculate the sample concentration, which is then multiplied by the dilution factor to obtain the actual concentration of the sample.

Figure S7 The standard curve of PPO

**8. TAL**

The kit assay Plant TAL level in the sample, use Purified Plant TAL antibody to coat microtiter plate wells, make solid-phase antibody, then add TAL to wells. Combined TAL antibody which With HRP labeled, become antibody-antigen-enzyme-antibody complex, after washing completely. Add TMB substrate solution, TMB substrate becomes blue color At HRP enzyme-catalyzed, reaction is terminated by the addition of a sulphuric acid solution and the color change is measured spectrophotometrically at a wavelength of 450 nm. The concentration of Plant TAL in the samples is then determined by comparing the OD of the samples to the standard curve.

Detection range, 16U/L~600U/L.

Table S15 Materials provided with the kit

| No. | Reagents | Specifications | No. | Reagents | Specifications |
| --- | --- | --- | --- | --- | --- |
| 1 | wash solution | 20mL×1 bottle | 7 | Stop Solution | 6mL×1 bottle |
| 2 | HRP-Conjugate reagent | 6mL×1 bottle | 8 | Standard (1200U/L) | 0.5mL×1 bottle |
| 3 | Microelisa stripplate | 12well×8strips | 9 | Standard diluent | 1.5mL×1 bottle |
| 4 | Sample diluent | 6mL×1 bottle | 10 | Instruction | 1 |
| 5 | Chromogen Solution A | 6mL×1 bottle | 11 | Closure plate membrane | 2 |
| 6 | Chromogen Solution B | 6mL×1 bottle | 12 | Sealed bags | 1 |

8.1 Assay procedure

(1) Preparation the standard solutions according to Table S16.

Table S16 Preparation of standard solution

| Concentration | No. | Procedure |
| --- | --- | --- |
| 600 U/L | 5 Standard | 150μL Original density Standard +150μL Standard diluent |
| 300 U/L | 4 Standard | 150μL 5 Standard +150μL Standard diluent |
| 150 U/L | 3 Standard | 150μL 4 Standard +150μL Standard diluent |
| 75 U/L | 2 Standard | 150μL 3 Standard +150μL Standard diluent |
| 37.5 U/L | 1 Standard | 150μL 2 Standard +150μL Standard diluent |

(2) Add sample

On the Microelisa stripplate, blank wells (blank control wells without samples or ELISA reagents, the rest of the steps are the same), standard wells, and wells for the sample to be tested were set separately. Accurately add 50 μL of each concentration standard solution to the standard wells. Add 40 μL of sample diluent to the sample wells, and then add 10 μL of the sample to be tested (the final dilution of the sample was 5 times).

(3) Incubate

After closing plate with Closure plate membrane, incubated for 30 min at 37℃.

(4) Configurate liquid

The wash solution diluted 30-fold with distilled water.

(5) Washing

Uncovered closure plate membrane, discard Liquid, dry by swing, add washing buffer to every well, still for 30s then drain. Repeat those process 5 times and pat dry.

(6) Add enzyme

Add HRP-Conjugate reagent 50μL to each well, except the blank well.

(7) Incubate

Same as procedure 3.

(8) Washing

Same as procedure 3.

(9) Color rendering

Add Chromogen Solution A 50μL and Chromogen Solution B to each well, evade the light preservation for 10 min at 37℃.

(10) Stop the reaction

Add Stop Solution 50μL to each well, at which point the blue color changed to yellow color.

(11) Determination

Take blank well as zero, read the absorbance (OD value) of each well at 450nm. The measurement should be conducted within 15 min after adding the stop solution.

8.2 Calculate

Draw a standard curve with the concentration of the standard substance as the x-axis and the OD value as the y-axis. Then, the linear regression equation (y=62.146x+1.5224, R^2^=0.9949) of the standard curve is calculated using the concentration and OD value of the standard substance. The OD value of the sample is substituted into the equation to calculate the sample concentration, which is then multiplied by the dilution factor to obtain the actual concentration of the sample.

Figure S8 The standard curve of TAL

**9. LOX**

The kit assay Plant LOX level in the sample, use Purified Plant LOX antibody to coat microtiter plate wells, make solid-phase antibody, then add LOX to wells. Combined LOX antibody which With HRP labeled, become antibody-antigen-enzyme-antibody complex, after washing completely. Add TMB substrate solution, TMB substrate becomes blue color At HRP enzyme-catalyzed, reaction is terminated by the addition of a sulphuric acid solution and the color change is measured spectrophotometrically at a wavelength of 450 nm. The concentration of Plant LOX in the samples is then determined by comparing the OD of the samples to the standard curve.

Detection range, 10U/L~340U/L.

Table S17 Materials provided with the kit

| No. | Reagents | Specifications | No. | Reagents | Specifications |
| --- | --- | --- | --- | --- | --- |
| 1 | wash solution | 20mL×1 bottle | 7 | Stop Solution | 6mL×1 bottle |
| 2 | HRP-Conjugate reagent | 6mL×1 bottle | 8 | Standard (640U/L) | 0.5mL×1 bottle |
| 3 | Microelisa stripplate | 12well×8strips | 9 | Standard diluent | 1.5mL×1 bottle |
| 4 | Sample diluent | 6mL×1 bottle | 10 | Instruction | 1 |
| 5 | Chromogen Solution A | 6mL×1 bottle | 11 | Closure plate membrane | 2 |
| 6 | Chromogen Solution B | 6mL×1 bottle | 12 | Sealed bags | 1 |

9.1 Assay procedure

(1) Preparation the standard solutions according to Table S18.

Table S18 Preparation of standard solution

| Concentration | No. | Procedure |
| --- | --- | --- |
| 320 U/L | 5 Standard | 150μL Original density Standard +150μL Standard diluent |
| 160 U/L | 4 Standard | 150μL 5 Standard +150μL Standard diluent |
| 80 U/L | 3 Standard | 150μL 4 Standard +150μL Standard diluent |
| 40 U/L | 2 Standard | 150μL 3 Standard +150μL Standard diluent |
| 20 U/L | 1 Standard | 150μL 2 Standard +150μL Standard diluent |

(2) Add sample

On the Microelisa stripplate, blank wells (blank control wells without samples or ELISA reagents, the rest of the steps are the same), standard wells, and wells for the sample to be tested were set separately. Accurately add 50 μL of each concentration standard solution to the standard wells. Add 40 μL of sample diluent to the sample wells, and then add 10 μL of the sample to be tested (the final dilution of the sample was 5 times).

(3) Incubate

After closing plate with Closure plate membrane, incubated for 30 min at 37℃.

(4) Configurate liquid

The wash solution diluted 30-fold with distilled water.

(5) Washing

Uncovered closure plate membrane, discard Liquid, dry by swing, add washing buffer to every well, still for 30s then drain. Repeat those process 5 times and pat dry.

(6) Add enzyme

Add HRP-Conjugate reagent 50μL to each well, except the blank well.

(7) Incubate

Same as procedure 3.

(8) Washing

Same as procedure 3.

(9) Color rendering

Add Chromogen Solution A 50μL and Chromogen Solution B to each well, evade the light preservation for 10 min at 37℃.

(10) Stop the reaction

Add Stop Solution 50μL to each well, at which point the blue color changed to yellow color.

(11) Determination

Take blank well as zero, read the absorbance (OD value) of each well at 450nm. The measurement should be conducted within 15 min after adding the stop solution.

9.2 Calculate

Draw a standard curve with the concentration of the standard substance as the x-axis and the OD value as the y-axis. Then, the linear regression equation (y=29.737x+0.3257, R^2^=0.9969) of the standard curve is calculated using the concentration and OD value of the standard substance. The OD value of the sample is substituted into the equation to calculate the sample concentration, which is then multiplied by the dilution factor to obtain the actual concentration of the sample.

Figure S9 The standard curve of LOX

**10. CI**

The kit assay Plant CI level in the sample, use Purified Plant CI antibody to coat microtiter plate wells, make solid-phase antibody, then add CI to wells. Combined CI antibody which With HRP labeled, become antibody-antigen-enzyme-antibody complex, after washing completely. Add TMB substrate solution, TMB substrate becomes blue color At HRP enzyme-catalyzed, reaction is terminated by the addition of a sulphuric acid solution and the color change is measured spectrophotometrically at a wavelength of 450 nm. The concentration of Plant CI in the samples is then determined by comparing the OD of the samples to the standard curve.

Detection range, 1.2ng/L~45 ng/L.

Table S19 Materials provided with the kit

| No. | Reagents | Specifications | No. | Reagents | Specifications |
| --- | --- | --- | --- | --- | --- |
| 1 | wash solution | 20mL×1 bottle | 7 | Stop Solution | 6mL×1 bottle |
| 2 | HRP-Conjugate reagent | 6mL×1 bottle | 8 | Standard (80ng/L) | 0.5mL×1 bottle |
| 3 | Microelisa stripplate | 12well×8strips | 9 | Standard diluent | 1.5mL×1 bottle |
| 4 | Sample diluent | 6mL×1 bottle | 10 | Instruction | 1 |
| 5 | Chromogen Solution A | 6mL×1 bottle | 11 | Closure plate membrane | 2 |
| 6 | Chromogen Solution B | 6mL×1 bottle | 12 | Sealed bags | 1 |

10.1 Assay procedure

(1) Preparation the standard solutions according to Table S20.

Table S20 Preparation of standard solution

| Concentration | No. | Procedure |
| --- | --- | --- |
| 40 ng/L | 5 Standard | 150μL Original density Standard +150μL Standard diluent |
| 20 ng/L | 4 Standard | 150μL 5 Standard +150μL Standard diluent |
| 10 ng/L | 3 Standard | 150μL 4 Standard +150μL Standard diluent |
| 5 ng/L | 2 Standard | 150μL 3 Standard +150μL Standard diluent |
| 2.5 ng/L | 1 Standard | 150μL 2 Standard +150μL Standard diluent |

(2) Add sample

On the Microelisa stripplate, blank wells (blank control wells without samples or ELISA reagents, the rest of the steps are the same), standard wells, and wells for the sample to be tested were set separately. Accurately add 50 μL of each concentration standard solution to the standard wells. Add 40 μL of sample diluent to the sample wells, and then add 10 μL of the sample to be tested (the final dilution of the sample was 5 times).

(3) Incubate

After closing plate with Closure plate membrane, incubated for 30 min at 37℃.

(4) Configurate liquid

The wash solution diluted 30-fold with distilled water.

(5) Washing

Uncovered closure plate membrane, discard Liquid, dry by swing, add washing buffer to every well, still for 30s then drain. Repeat those process 5 times and pat dry.

(6) Add enzyme

Add HRP-Conjugate reagent 50μL to each well, except the blank well.

(7) Incubate

Same as procedure 3.

(8) Washing

Same as procedure 3.

(9) Color rendering

Add Chromogen Solution A 50μL and Chromogen Solution B to each well, evade the light preservation for 10 min at 37℃.

(10) Stop the reaction

Add Stop Solution 50μL to each well, at which point the blue color changed to yellow color.

(11) Determination

Take blank well as zero, read the absorbance (OD value) of each well at 450nm. The measurement should be conducted within 15 min after adding the stop solution.

10.2 Calculate

Draw a standard curve with the concentration of the standard substance as the x-axis and the OD value as the y-axis. Then, the linear regression equation (y=3.6205x+0.0579, R^2^=0.9971) of the standard curve is calculated using the concentration and OD value of the standard substance. The OD value of the sample is substituted into the equation to calculate the sample concentration, which is then multiplied by the dilution factor to obtain the actual concentration of the sample.

Figure S10 The standard curve of CI

**11. TI**

The kit assay Plant TI level in the sample, use Purified Plant TI antibody to coat microtiter plate wells, make solid-phase antibody, then add TI to wells. Combined TI antibody which With HRP labeled, become antibody-antigen-enzyme-antibody complex, after washing completely. Add TMB substrate solution, TMB substrate becomes blue color At HRP enzyme-catalyzed, reaction is terminated by the addition of a sulphuric acid solution and the color change is measured spectrophotometrically at a wavelength of 450 nm. The concentration of Plant TI in the samples is then determined by comparing the OD of the samples to the standard curve.

Detection range, 1 ng/L~50 ng/L.

Table S21 Materials provided with the kit

| No. | Reagents | Specifications | No. | Reagents | Specifications |
| --- | --- | --- | --- | --- | --- |
| 1 | wash solution | 20mL×1 bottle | 7 | Stop Solution | 6mL×1 bottle |
| 2 | HRP-Conjugate reagent | 6mL×1 bottle | 8 | Standard (80ng/L) | 0.5mL×1 bottle |
| 3 | Microelisa stripplate | 12well×8strips | 9 | Standard diluent | 1.5mL×1 bottle |
| 4 | Sample diluent | 6mL×1 bottle | 10 | Instruction | 1 |
| 5 | Chromogen Solution A | 6mL×1 bottle | 11 | Closure plate membrane | 2 |
| 6 | Chromogen Solution B | 6mL×1 bottle | 12 | Sealed bags | 1 |

11.1 Assay procedure

(1) Preparation the standard solutions according to Table S22.

Table S22 Preparation of standard solution

| Concentration | No. | Procedure |
| --- | --- | --- |
| 40 ng/L | 5 Standard | 150μL Original density Standard +150μL Standard diluent |
| 20 ng/L | 4 Standard | 150μL 5 Standard +150μL Standard diluent |
| 10 ng/L | 3 Standard | 150μL 4 Standard +150μL Standard diluent |
| 5 ng/L | 2 Standard | 150μL 3 Standard +150μL Standard diluent |
| 2.5 ng/L | 1 Standard | 150μL 2 Standard +150μL Standard diluent |

(2) Add sample

On the Microelisa stripplate, blank wells (blank control wells without samples or ELISA reagents, the rest of the steps are the same), standard wells, and wells for the sample to be tested were set separately. Accurately add 50 μL of each concentration standard solution to the standard wells. Add 40 μL of sample diluent to the sample wells, and then add 10 μL of the sample to be tested (the final dilution of the sample was 5 times).

(3) Incubate

After closing plate with Closure plate membrane, incubated for 30 min at 37℃.

(4) Configurate liquid

The wash solution diluted 30-fold with distilled water.

(5) Washing

Uncovered closure plate membrane, discard Liquid, dry by swing, add washing buffer to every well, still for 30s then drain. Repeat those process 5 times and pat dry.

(6) Add enzyme

Add HRP-Conjugate reagent 50μL to each well, except the blank well.

(7) Incubate

Same as procedure 3.

(8) Washing

Same as procedure 3.

(9) Color rendering

Add Chromogen Solution A 50μL and Chromogen Solution B to each well, evade the light preservation for 10 min at 37℃.

(10) Stop the reaction

Add Stop Solution 50μL to each well, at which point the blue color changed to yellow color.

(11) Determination

Take blank well as zero, read the absorbance (OD value) of each well at 450nm. The measurement should be conducted within 15 min after adding the stop solution.

11.2 Calculate

Draw a standard curve with the concentration of the standard substance as the x-axis and the OD value as the y-axis. Then, the linear regression equation (y=4.2678x+0.0212, R^2^=0.9959) of the standard curve is calculated using the concentration and OD value of the standard substance. The OD value of the sample is substituted into the equation to calculate the sample concentration, which is then multiplied by the dilution factor to obtain the actual concentration of the sample.

Figure S11 The standard curve of TI

**12. JA**

The kit assay Plant JA level in the sample, use Purified Plant JA antibody to coat microtiter plate wells, make solid-phase antibody, then add JA to wells. Combined JA antibody which With HRP labeled, become antibody-antigen-enzyme-antibody complex, after washing completely. Add TMB substrate solution, TMB substrate becomes blue color At HRP enzyme-catalyzed, reaction is terminated by the addition of a sulphuric acid solution and the color change is measured spectrophotometrically at a wavelength of 450 nm. The concentration of Plant JA in the samples is then determined by comparing the OD of the samples to the standard curve.

Detection range, 30pmol/L~1000pmol/L.

Table S23 Materials provided with the kit

| No. | Reagents | Specifications | No. | Reagents | Specifications |
| --- | --- | --- | --- | --- | --- |
| 1 | wash solution | 20mL×1 bottle | 7 | Stop Solution | 6mL×1 bottle |
| 2 | HRP-Conjugate reagent | 6mL×1 bottle | 8 | Standard (2000pmol/L) | 0.5mL×1 bottle |
| 3 | Microelisa stripplate | 12well×8strips | 9 | Standard diluent | 1.5mL×1 bottle |
| 4 | Sample diluent | 6mL×1 bottle | 10 | Instruction | 1 |
| 5 | Chromogen Solution A | 6mL×1 bottle | 11 | Closure plate membrane | 2 |
| 6 | Chromogen Solution B | 6mL×1 bottle | 12 | Sealed bags | 1 |

12.1 Assay procedure

(1) Preparation the standard solutions according to Table S24.

Table S24 Preparation of standard solution

| Concentration | No. | Procedure |
| --- | --- | --- |
| 1000pmol/L | 5 Standard | 150μL Original density Standard +150μL Standard diluent |
| 500pmol/L | 4 Standard | 150μL 5 Standard +150μL Standard diluent |
| 250pmol/L | 3 Standard | 150μL 4 Standard +150μL Standard diluent |
| 125pmol/L | 2 Standard | 150μL 3 Standard +150μL Standard diluent |
| 62.5pmol/L | 1 Standard | 150μL 2 Standard +150μL Standard diluent |

(2) Add sample

On the Microelisa stripplate, blank wells (blank control wells without samples or ELISA reagents, the rest of the steps are the same), standard wells, and wells for the sample to be tested were set separately. Accurately add 50 μL of each concentration standard solution to the standard wells. Add 40 μL of sample diluent to the sample wells, and then add 10 μL of the sample to be tested (the final dilution of the sample was 5 times).

(3) Incubate

After closing plate with Closure plate membrane, incubated for 30 min at 37℃.

(4) Configurate liquid

The wash solution diluted 30-fold with distilled water.

(5) Washing

Uncovered closure plate membrane, discard Liquid, dry by swing, add washing buffer to every well, still for 30s then drain. Repeat those process 5 times and pat dry.

(6) Add enzyme

Add HRP-Conjugate reagent 50μL to each well, except the blank well.

(7) Incubate

Same as procedure 3.

(8) Washing

Same as procedure 3.

(9) Color rendering

Add Chromogen Solution A 50μL and Chromogen Solution B to each well, evade the light preservation for 10 min at 37℃.

(10) Stop the reaction

Add Stop Solution 50μL to each well, at which point the blue color changed to yellow color.

(11) Determination

Take blank well as zero, read the absorbance (OD value) of each well at 450nm. The measurement should be conducted within 15 min after adding the stop solution.

12.2 Calculate

Draw a standard curve with the concentration of the standard substance as the x-axis and the OD value as the y-axis. Then, the linear regression equation (y=113.36x+0.4166, R^2^=0.9952) of the standard curve is calculated using the concentration and OD value of the standard substance. The OD value of the sample is substituted into the equation to calculate the sample concentration, which is then multiplied by the dilution factor to obtain the actual concentration of the sample.

Figure S12 The standard curve of JA
