# Supplementary material for: Alfalfa leaf weevil larvae and adults feeding induces physiological change in defensive enzymes of alfalfa
Source: PLoS One. 2024 Nov 8;19(11):e0312612. doi: 10.1371/journal.pone.0312612 (PMC11548782; doi:10.1371/journal.pone.0312612)
Supplement: Attachment — Submitted filename: Response to Reviewers.docx [file pone.0312612.s003.docx]

Journal Requirements:

1. When submitting your revision, we need you to address these additional requirements.

Please ensure that your manuscript meets PLOS ONE's style requirements, including those for file naming. The PLOS ONE style templates can be found at 
https://journals.plos.org/plosone/s/file?id=wjVg/PLOSOne_formatting_sample_main_body.pdf and 
<https://journals.plos.org/plosone/s/file?id=ba62/PLOSOne_formatting_sample_title_authors_affiliations.pdf>

Response: We have completed the formatting revision of the manuscript according to the requirements of the PLOS ONE, which fully meets the style of the PLOS ONE.

2. Thank you for stating the following financial disclosure: " National Modern Agriculture Industry Technology System (CARS-34) of the Ministry of Agriculture and Rural Affairs".

Please state what role the funders took in the study.  If the funders had no role, please state: "The funders had no role in study design, data collection and analysis, decision to publish, or preparation of the manuscript."

If this statement is not correct you must amend it as needed.

Please include this amended Role of Funder statement in your cover letter; we will change the online submission form on your behalf.

Response: We had added the Role of Funder statement in our cover letter.

3. PLOS requires an ORCID iD for the corresponding author in Editorial Manager on papers submitted after December 6th, 2016. Please ensure that you have an ORCID iD and that it is validated in Editorial Manager. To do this, go to ‘Update my Information’ (in the upper left-hand corner of the main menu), and click on the Fetch/Validate link next to the ORCID field. This will take you to the ORCID site and allow you to create a new iD or authenticate a pre-existing iD in Editorial Manager. Please see the following video for instructions on linking an ORCID iD to your Editorial Manager account: <https://www.youtube.com/watch?v=_xcclfuvtxQ>

Response: I have completed the registration and updated my personal information in the system. ORCID iD is 0009-0005-7290-0600.

4. Please include captions for your Supporting Information files at the end of your manuscript, and update any in-text citations to match accordingly. Please see our Supporting Information guidelines for more information: http://journals.plos.org/plosone/s/supporting-information.

Response: Our supporting information is the method for measuring enzyme activity, which has been supplemented at the end of the main text.

Additional Editor Comments:

The manuscript described about the feeding activity of alfalfa leaf weevil larvae and adults, and physiological changes in alfalfa. The study design and manuscript preparation was well. Study background information, objective and methodology was clearly described in the manuscript. However, results and interpretations sections need revision. Authors needs to consider the following comments to improve the manuscript.

Response: Thank you for your positive feedback and for raising this important point. We had carefully revised the entire text and further improved the writing quality of the manuscript.

Line 29: In addition to cow, add two more examples.

Response: Thank you for the constructive feedback. We had added more examples.

Line 105: Write expansion of PBS.
Response: Thank you for your comments and suggestions on our manuscript. We had added revised it.

Line 426: Remove “[34]”

Response: Thank you for the constructive feedback. We had added removed it.

Methodology for each enzyme assay may describe in the methodology section instead of providing as supplementary section. Supportive tables/figures/details may provide as supplementary information.

Response: Thank you for your insightful comment. The enzyme activity assays were conducted using reagent kits, and the reagents in each kit for enzyme activity vary greatly. If all methods are included in the main text, it will increase the length of the text, so we have included them in the supporting information.

Add methodology for Principal Component analysis.

Response: The principal component analysis method has been added to the “Data Statistics and Processing” section.

In section 3.1. and 3.2.: Avoid repetitions of “in alfalfa leaves induced by adult alfalfa leaf weevils” and “in alfalfa leaves induced by 3rd-instar larvae of alfalfa leaf Weevils” in subsection titles.

Response: Thank you for your valuable suggestion. We had added revised it.

In Figure 2 - 5: Remove abbreviations in Y axis titles.

Response: Thank you for your comments and suggestions on our manuscript. We had removed the abbreviations in Y axis titles of Fig 2-5.

In Figure 6 – 7 & 12 - 13: Write expansions for all the abbreviations in the figure title.

Response: Thank you for your comments and suggestions on our manuscript. In Figure 6 – 7 & 12 – 13, the abbreviations in the figures have appeared multiple times in the main text, and we provided full spelling for each abbreviation when it first appeared. Therefore, we believe it is not necessary to annotate the full spelling of abbreviations in the figure title.

In Figures: Results are expressed in enzyme specific activity based units. Whereas in the text, results were described in percentage levels. Please check and describe clearly with respect to the presented results or add additional informations for better understanding.

Response: The percentage comparison is the result of comparing enzyme specific activity based units, without any special calculation.

Please check the cited references with the reference list vice versa. Check and write all the references as per the journal guidelines. For example, see line 507-508, 526-527, 546, 547, etc.,

Response: Thank you for your comments and suggestions on our manuscript. We had revised it.

Please check the typographical errors throughout the manuscript.
Response: Thank you for your comments and suggestions on our manuscript. We had revised it.

5. Review Comments to the Author

Reviewer #1: Cohesive, beautifully structured MS. The choice of topic is also an economically important field, but the stress physiology of insect damage to alfalfa is also important in basic research. The methods and implementation of the experimental design and research work are of scientific standard. The text of the thesis is easy to follow, its structure is based on a nice internal logic. Perhaps some of the groundwork ideas from Discussion could have been included in Introduction. In evaluating the results, correlations are sought and found. Unfortunately, many sentences in the conclusions chapters are identical to some sentences in the abstract and discussion chapters. Please check this for the other quote species as well.
Response: Thank you for your positive feedback and for raising this important point. We had carefully revised the entire text and further improved the writing quality of the manuscript.

Other comments:
ad 89-90: Do I need a company name? Hoagland solution is well-known, easy to prepare, widely used.

Response: Thank you for your valuable feedback. We had deleted it.

ad 160 & 284: Proposed simplification: metabolic enzymes

Response: Thank you for your insightful comment. We had revised it.

ad 190: Is this citation really relevant ?

Response: Thank you for the constructive feedback. We deleted that citation.

ad 198: TI or TPI?

Response: Thank you for your kindly suggestions. It should be “TI”, we had revised it.

AD 367/375/412/415: It is not customary to put the species descriptive auctor in parentheses. It is enough to specify the auctor at the first summons. Alternatively, it should be given uniformly, i.e. for the other species (see Frankliniella occidentalis in line 67). Please check this for the other quoted species as well.

Response: Thank you for your kindly suggestions. We had deleted the species descriptive.

ad 430: read: Paspalum dilatatum

Response: Thank you for your kindly suggestions. We had revised it.

Not completed citations: 550-551 (PhD Thesis?), 555-556, 575-576
Response: Yes, it is PhD thesis.

Please write in Italic: 532
Response: Thank you for your kindly suggestions. We had revised it.

Typing and character errors (extra space etc.) : 31, 39, 420, 425, 511, 514, 520,

Response: I am very sorry for the mistake, we had revised it.

Reviewer #2: Very nice work to identify the enzymes involved in response to alfalfa weevil feeding
Point wise comments and suggestions given on attached manuscript

Response: Thank you for your valuable feedback.

Regression or correlation matrix table might be much better to understand when in numbers
Numerical data would be much easy to understand if tables of the figures are presented and AVOVA for overall enzymes of respective group may be presented instead of figure for each to identify the impact of factors separately and in interaction either in manuscript or in supplementary file

Response: Thank you for your kindly suggestions. We had revised it in Fig 6 and Fig 12.

The percentage comparison calculation may also be presented in method as well. Its overall correlation can also help for better relate the things or parameters in focus

Response: The percentage comparison is the result of comparing enzyme specific activity based units, without any special calculation.

Line 426 Need to follow format of the journal.

Response: We have completed the formatting revision of the manuscript according to the requirements of the PLOS ONE, which fully meets the style of the PLOS ONE.
