# Supplementary material for: Alfalfa leaf weevil larvae and adults feeding induces physiological change in defensive enzymes of alfalfa
Source: PLoS One. 2024 Nov 8;19(11):e0312612. doi: 10.1371/journal.pone.0312612 (PMC11548782; doi:10.1371/journal.pone.0312612)
Supplement: Attachment — Submitted filename: Response to Reviewers.docx [file pone.0312612.s004.docx]

**Journal Requirements:**
Please review your reference list to ensure that it is complete and correct. If you have cited papers that have been retracted, please include the rationale for doing so in the manuscript text, or remove these references and replace them with relevant current references. Any changes to the reference list should be mentioned in the rebuttal letter that accompanies your revised manuscript. If you need to cite a retracted article, indicate the article’s retracted status in the References list and also include a citation and full reference for the retraction notice.
**Response:** Thank you for the constructive feedback. In our manuscript, we cited two Chinese doctoral dissertations. We have traced their published English articles and replaced.

**Additional Editor Comments:**
Line 4-5: Manuscript title should be specific to the study. Authors may write “defensive enzymes of alfalfa” instead of “alfalfa enzymes” in the title.
**Response:** Thank you for the constructive feedback. We had replaced “alfalfa enzymes” with “defensive enzymes of alfalfa” in the title.

Line 32-33: Remove the sentence “Adults feed on alfalfa stems and leaves, whereas larvae feed on leaf flesh and veins.”.
**Response:** Thank you for your comments and suggestions on our manuscript. We had removed that sentence.

Line 33-34 & 476-478: The study conclusion is not clear. Please check “PPO is distributed in flower organs, meristems, leaves, stems, and roots ………”. Among the examined all enzymes, how PPO only played important role?. Please justify with other enzymes or write conclusion in comparative way with other enzymes.
**Response:** Thank you for your valuable feedback. To avoid ambiguity, we have removed this sentence.

Line 67: Is it “Phenylalaninammo nialyase” or “Phenylalanine ammonia lyase”?. Please check.
**Response:** Thank you for your valuable feedback. It should be “phenylalanine ammonia-lyase”, we had revised it.

Line 73-74: Write common of Frankliniella occidentalis.
**Response:** Thank you for your valuable feedback. We had revised it.

Line 88-89: Remove the sentence “This study provides a reference basis for research on alfalfa insect resistance mechanisms and breeding.” from the study objective.
**Response:** Thank you for your valuable feedback. We had deleted it.

Line 119-120: Write like “A. Adult feeding, B. Larvae feeding” instead of “A for adult and B for larvae”. Remove “(replicates)” from the figure legend.
**Response:** Thank you for the constructive feedback. We had revised it.

Line 125-126: Add methodology for Principal Component analysis. The sentence “The perform correlation analysis using Pearson correlation coefficient.” is not clear. Please check and revise.
**Response:** Thank you for the constructive feedback. The principal component analysis method has been added to the “Data Statistics and Processing” section. “Perform principal component analysis using Pearson correlation coefficient.”

Reviewers' comments:

**Reviewer #1:** Comments and suggestions from reviewers have been accepted, but not always improved (e.g. Wu 2022, Ren 2020, Malefo et al. 2020), although these changes may be subject to editorial decision.

**Response:** Thank you for the constructive feedback. In our manuscript, we cited two Chinese doctoral dissertations. We have traced their published English articles and replaced.
